# Prokaryotes in Subsoil—Evidence for a Strong Spatial Separation of Different Phyla by Analysing Co-occurrence Networks

**DOI:** 10.3389/fmicb.2015.01269

**Published:** 2015-11-18

**Authors:** Marie Uksa, Michael Schloter, David Endesfelder, Susanne Kublik, Marion Engel, Timo Kautz, Ulrich Köpke, Doreen Fischer

**Affiliations:** ^1^Research Unit Environmental Genomics, Department of Environmental Science, Helmholtz Zentrum MünchenOberschleissheim, Germany; ^2^Scientific Computing Research Unit, Institute of Computational Biology, Helmholtz Zentrum MünchenOberschleissheim, Germany; ^3^Institute of Organic Agriculture, University of BonnBonn, Germany

**Keywords:** subsoil, drilosphere, rhizosphere, bacterial diversity, core microbiome, co-occurrence

## Abstract

Microbial communities in soil provide a wide range of ecosystem services. On the small scale, nutrient rich hotspots in soil developed from the activities of animals or plants are important drivers for the composition of microbial communities and their functional patterns. However, in subsoil, the spatial heterogeneity of microbes with differing lifestyles has been rarely considered so far. In this study, the phylogenetic composition of the bacterial and archaeal microbiome based on 16S rRNA gene pyrosequencing was investigated in the soil compartments bulk soil, drilosphere, and rhizosphere in top- and in the subsoil of an agricultural field. With co-occurrence network analysis, the spatial separation of typically oligotrophic and copiotrophic microbes was assessed. Four bacterial clusters were identified and attributed to bulk topsoil, bulk subsoil, drilosphere, and rhizosphere. The bacterial phyla Proteobacteria and Bacteroidetes, representing mostly copiotrophic bacteria, were affiliated mainly to the rhizosphere and drilosphere—both in topsoil and subsoil. Acidobacteria, Actinobacteria, Gemmatimonadetes, Planctomycetes, and Verrucomicrobia, bacterial phyla which harbor many oligotrophic bacteria, were the most abundant groups in bulk subsoil. The bacterial core microbiome in this soil was estimated to cover 7.6% of the bacterial sequencing reads including both oligotrophic and copiotrophic bacteria. In contrast the archaeal core microbiome includes 56% of the overall archaeal diversity. Thus, the spatial variability of nutrient quality and quantity strongly shapes the bacterial community composition and their interaction in subsoil, whereas archaea build a stable backbone of the soil prokaryotes due to their low variability in the different soil compartments.

## Introduction

Soils are known as hotspots for biodiversity. Moreover soils provide a wide range of ecosystem services including nutrient cycling, carbon sequestration, safeguarding of water resources and plant growth promotion (van der Heijden et al., 2008; Berg, 2009; Bardgett and van der Putten, 2014). In contrast to the microbiome of topsoil, which has been well studied in the last decades, focussing on microbial community structure and function as well as plant-microbe interactions (Berg and Smalla, 2009), microbes below the plow horizon so far are poorly investigated. The general opinion implies a decrease of abundance, diversity and activity of bacteria, fungi and archaea with soil depth as a result of the more oligotrophic conditions present in deeper soil layers; consequently it is assumed that the contribution of the subsoil microbiome to the overall turnover of nutrients in soil is low (Fuka et al., 2009; Eilers et al., 2012; Stone et al., 2014). However, these observations are biased by the fact that small-scale spatial heterogeneity of microbes in subsoils has received almost no attention and the presence of hotspots in subsoils, which may change the described low microbial activity in subsoils, has been mostly overlooked (Nunan et al., 2003; Vos et al., 2013).

Commonly hotspots in subsoils are mainly connected to vertical biopores, which are formed by earthworms or thick tap roots (Kuzyakov and Blagodatskaya, 2015). These biopores are characterized by relatively high nutrient input due to plant exudates in the rhizosphere (Neumann et al., 2014) or cast deposition of earthworms and their coating in the drilosphere (Andriuzzi et al., 2013). As microbial community composition is linked to substrate quantity and quality (Marschner et al., 2001; Aira et al., 2010; Stromberger et al., 2012) a pronounced spatial heterogeneity of microbes with differing lifestyle in subsoils can be assumed. This has been partly confirmed by DNA based fingerprint analyses of bacterial community structure. Here differences between bulk soil, drilosphere, and rhizosphere communities in subsoil were more pronounced in subsoil compared to topsoil (Uksa et al., 2014). These differences induced a high spatial variability of potential enzyme activities in the investigated subsoil compartments (Uksa et al., 2015). However, still data is missing on microbial network structures in the different subsoil compartments and the related ecophysiology of the microbiomes.

In this study we analyzed archaeal and bacterial community composition based on barcoding of 16S rRNA after PCR amplification of DNA directly extracted from bulk soil, drilosphere and rhizosphere of top- and subsoil samples from an agricultural field planted with the fodder crop *Cichorium intybus*. This plant species is known to strongly structure soils by the formation of thick biopores also in the subsoil (Löfkvist et al., 2005; Kautz et al., 2014). We analyzed network structures and co-occurrence pattern in the different compartments. We adressedthe question whether for top- and subsoils a specific set of co-occurring microbes can be identified independent from the spatial variability in each soil layer or if each hotspot (rhizosphere or drilosphere) harbors a set of co-occuring microbes independent from soil depth. The latter would emphasize a selection of microbiomes by earthworms or plants (Berg and Smalla, 2009). In addition the number of shared microbes and the size of the core microbiome in topsoil and subsoil was estimated. Based on our previous results (Uksa et al., 2014) we hypothesized that in topsoils the number of shared OTUs between the different compartments bulk soil, drilosphere and rhizosphere is higher as compared to subsoils.

## Materials and methods

### Experimental field site and soil sampling

Soil samples were obtained from three separated plots (each 10 × 6 m) of an agricultural field at Klein-Altendorf (Germany; 50°37′21” N, 6°59′29” E) in May 2011 and treated as true replicates. At the month of sampling the mean temperature was 14.8°C and mean daily precipitation was 33.2 mm (Agrarmeteorologie Rheinland-Pfalz; www.wetter.rlp.de). *Cichorium intybus* L. was grown on the field for the third year; at the sampling time point plants were in the early flowering stage. *C. intybus* has a tap root system and thus forms large sized biopores, which significantly structure the soil (Löfkvist et al., 2005). The soil has been classified as Haplic Luvisol and characterized by a silty clay loam texture with clay accumulation in the subsoil between 45 and 95 cm (Gaiser et al., 2012).

For soil sampling, one soil pit per plot with a size of 1 × 1 × 1 m was excavated using a hydraulic shovel. Before sampling about 5 cm per side wall were carefully removed by a spade. From the profiles, the bulk soil, the drilosphere and the rhizosphere were sampled both in topsoil (10–30 cm) and subsoil (60–75 cm). One millimeter coatings around earthworm burrows of 0.4–1.2 cm were considered as drilosphere and scraped out with a small sterile spoon. Roots were sampled from the soil profiles together with maximal 2 mm adhering soil by using sterile tweezers. The adhering soil was referred as rhizosphere. Soil with no roots and earthworm channels was defined as bulk soil. At least 5 subsamples for each compartment were pooled from each profile, transported on dry ice and stored at −80°C before DNA extraction.

### DNA extraction and quantitative real-time PCR of 16S rRNA genes

DNA was extracted using the FastDNA® Spin Kit for Soil (MP Biomedicals, Eschwege, Germany) following the manufacturer's protocol. To enhance DNA yield, an additional bead beating step for 40 s and an incubation step at 55°C for 5 min before elution was performed. NanoDrop 1000 Spectrophotometer (PeqLab, Erlangen, Germany) was used for DNA quality assessment by measurement A_260nm_/A_280nm_ and A_260nm_/A_230nm_ ratios. The DNA concentration was determined from 250-fold dilutions using the Quant-iT™ PicoGreen® dsDNA Assay Kit (Life Technologies, Darmstadt, Germany) with a detection range from 0.016 to 1 ng·μl^−1^.

Abundance of bacterial and archaeal 16S rRNA genes was quantified by real-time PCR using a 7300 Real-Time PCR System (Applied Biosystems, Darmstadt, Germany) and the Power SYBR® Green PCR Master Mix (Applied Biosystems) following the protocol described by Töwe et al. (2010). Primers rSAf(i) (Nicol et al., 2003) and 985r (Bano et al., 2004) were used for archaeal 16S rRNA gene amplification, whereas bacterial 16S rRNA gene copy numbers were quantified with primers FP16S and RP16S (Bach et al., 2002) at a final concentration of 0.2 or 0.4 μM, respectively. According to an *in silico* analysis using the Genomatix software, version November 2012 (www.genomatrix.de), the archaeal primer pair covered representatives of Thaumarchaeota, Euryarchaeota and Crenarchaeota and thus could be considered as universal. Cloned 16S rRNA genes from *Methanobacterium* sp. (Timmers et al., 2012) and *Clavibacter michiganensis* subsp. *michiganensis* (DSM 46364) were used as qPCR standards for archaea and bacteria respectively. The DNA template was 128-fold diluted to avoid inhibition as tested in pre-experiments (data not shown). To increase efficiency of archaeal real-time PCR, 0.06% BSA was added to the master mix. For the amplification of bacterial 16S rRNA genes 40 PCR cycles (95°C—20 s, 62°C—1 min, 72°C—30 s) were performed; for amplification of the archaeal 16S rRNA genes 5 PCR cycles (95°C—20 s, 55°C—1 min, 72°C—30 s, lowering the annealing temperature for 1°C each cycle) followed by 35 PCR cycles with 50°C annealing temperature were performed. PCR efficiency was 85% for archaeal, and 92% for bacterial 16S rRNA gene amplification.

### Barcoded pyrosequencing and data processing

PCR amplicons of bacterial and archaeal 16S rRNA genes were sequenced using the 454 GS FLX+ instrument (Roche, Penzberg, Germany) following the manufacturer's protocols for amplicon library preparation (version June 2013) and emPCR amplification (version May 2011) with primers for unidirectional sequencing (Lib-L) and the XL+ Kit (version June 2013).

The specific primer sequences for bacterial 16S rRNA genes were 27F (5′-AGAGTTTGATCMTGGCTC-3′; *E. coli* position 8-25; Lane, 1991) and 984r (5′-GTAAGGTTCYTCGCG-3′; *E. coli* position 970-985; Klindworth et al., 2013). For archaeal 16S rRNA genes, the primer pair rSAf(i) (5′-CCTAYGGGGCGCAGCAG-3′; *E. coli* position 341–357; Nicol et al., 2003) and 958r (5′-YCCGGCGTTGAMTCCAATT-3′; *E. coli* position 940–958; Bano et al., 2004) was used.

Following the 454 sequencing guidelines for unidirectional sequencing, primer sequences were extended by the adapter sequences A and B for forward and reverse primers respectively; in addition the forward primer was labeled with a multiplex indices (MID). PCR reaction was performed with FastStart™ High Fidelity PCR System (Roche). To improve PCR efficiency 0.3% BSA was added; for the amplification of the archaeal 16S rRNA gene in addition 8% DMSO, as suggested by Timmers et al. (2012), was added. For amplification 1 ng DNA (bacterial 16S rRNA gene) respectively 30 ng (archaeal 16S rRNA gene) was used as template. PCR was initiated by a heating step to 95°C for 5 min followed by 25 (bacterial 16S rRNA gene) respectively 30 (archaeal 16S rRNA gene) cycles (95°C for 1 min, 50°C for 1 min and 72°C for 1 min) followed by a final extension at 72°C for 10 min.

Three PCR amplicons for each sample were pooled and purified with the NucleoSpin® Gel and PCR cleanup Kit (MACHEREY-NAGEL, Düren, Germany). The final DNA amount of the amplicon libraries was determined with Quant-iT™ PicoGreen® dsDNA Assay Kit as mentioned above. The average fragment size was measured with Agilent 2100 bioanalyzer instrument using the Agilent DNA 7500 Kit (Agilent Technologies, Waldbronn, Germany). The final sequencing run was performed according to the manufacturer's protocol and initial data processing was performed using gsRunProcessor v2.9.

Data processing of raw flowgrams was carried out with mothur (release v.1.33.0; Schloss et al., 2009) following the 454 SOP by Schloss et al. (2011). The SILVA reference file, comprising of bacterial, archaeal, and eukaryotic rRNA sequences of the small subunit (release 119; Quast et al., 2013) was used for alignment and chimera removal. Sequences were classified with the RDP database (release 10; Cole et al., 2014), which included both bacterial and archaeal 16S rRNA sequences, at 80% confidence level. OTUs were assigned by clustering at 95 and 90% similarity level. Pyrosequencing data sets were deposited at GenBank's Short Read Archive under the following accession number: PRJNA293151 (BioProject).

### Statistical analyses

Statistical analysis and graphic illustrations were computed with the R software (version 3.0.2; R Core Team, 2013) and the packages “agricolae” (de Mendiburu, 2014), “scatterplot3d” (Ligges and Mächler, 2003), “shape” (Soetaert, 2014), “stats” (R Core Team, 2013), “vcd” (Meyer et al., 2014), and “vegan” (Oksanen et al., 2015). Reads were subsampled according to the minimum number of reads per sample (3081 archaeal/4815 bacterial sequences). Richness, rarefaction and Shannon diversity index were calculated on the basis of 90% similarity level, as rarefractures analysis indicated full coverage at this level. As the “species” definition of prokaryotes at =97% similarity is still a controversial topic and RDP database classifies OTUs only down to 95% which corresponds to the genus level, all other analyses were performed on this similarity level. Prior to multivariate analysis with PerMANOVA, relative abundance data was Hellinger-transformed (Ramette, 2007). Significant differences within single OTUs were tested by ANOVA followed by posthoc Tukey-HSD test (α = 0.05). Bonferroni test was used for adjustment of *P*-values after multiple comparisons.

As the copy number of 16S rRNA genes highly varies across bacterial and archaeal genomes, 16S rRNA gene abundance data was adjusted according to the Ribosomal RNA Database (*rrn*DB; Stoddard et al., 2014) by using the “Pan-taxa statistics for RDP taxonomy” file (release 4.3.3). To obtain the adjusted abundance for each OTU, the absolute abundance of 16S rRNA reads were divided by the mean copy number of 16S rRNA genes per genome for the corresponding genus or nearest classifiable level. The resulting discrepancy between 16S rRNA gene abundance and adjusted abundance in every sequenced sample was used to correct the total 16S rRNA gene abundance determined by qPCR.

All OTUs with a minimum of 6 reads in at least 3 samples were considered for the estimation of microbial co-occurrence networks. Co-occurrence between any pair of OTUs was defined by a significant correlation (*P* < 0.05) with a correlation coefficient >0.6. The corresponding co-occurrence network was derived by setting an edge between pairs of co-occurring OTUs. To analyse spurious correlations caused by the compositional structure of the relative abundance, the CCREPE (Faust et al., 2012) method was used to estimate *P*-values from Spearman's rank correlation coefficients. Clusters of co-occurring OTUs were defined from the resulting co-occurrence network by grouping OTUs with high intra-cluster connectivity and low connectivity to other OTU clusters. Microbial clusters were identified by using the Markov Dynamics clustering algorithm (Schaub et al., 2012) implemented in MATLAB®. This algorithm allowed the identification of clique-like communities within a continuous range of a parameter (i.e., Markov time), capturing dynamic characteristics of processes on the network. The number of clusters of co-occurring OTUs was determined by choosing a community number larger than two which had the longest stable assignment over a range of Markov time points. Similarly to positive correlations, OTUs were defined to be negatively correlated if the correlation coefficient was < −0.6.

## Results

### Abundance of 16S rRNA genes from archaea and bacteria in different soil compartments of top- and subsoil

Microbial bbiomass was estimated by the amount of extracted DNA and related to soil dry weight (Figure S1; Gangneux et al., 2011). As expected, highest amounts of DNA were extracted from rhizosphere samples; DNA concentrations in the drilosphere were lower but still higher than in bulk soil (*P* = 0.001). Whereas no significant differences were found in DNA concentrations comparing rhizosphere samples from the top- and the subsoil, for bulk soil and drilosphere significant lower DNA concentrations were measured in subsoil as compared to topsoil (*P* = 0.005, *P* = 0.011).

**Figure 1:**
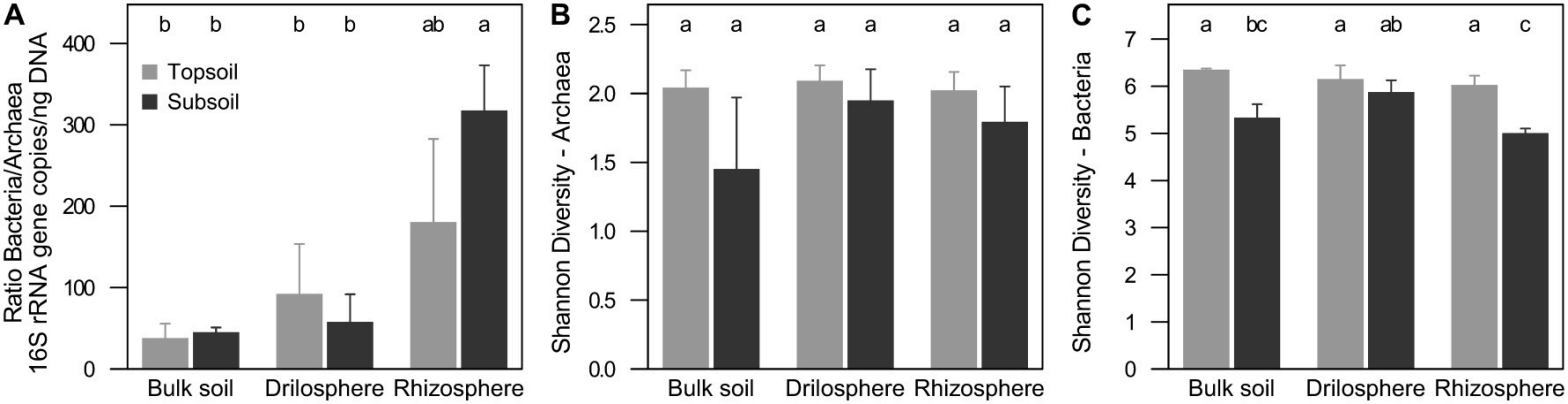
**Relation of 16S rRNA copies between archaea and bacteria (A) and Shannon diversity index at 90% similarity level of archaea (B) and bacteria (C) in soil compartments of topsoil and subsoil**. Different letters indicate significant differences (*P* ≤ 0.05).

QPCR analysis revealed 10^7^-10^9^ archaeal and 10^8^-10^12^ bacterial 16S rRNA copies g^−1^ dry weight. For all soil compartments in topsoil and subsoil, bacterial 16S rRNA gene copy numbers were higher compared to their archaea counterpart (*P* < 0.001). Ratios of bacterial to archaeal 16S rRNA gene copy numbers were in the range of 20–380 (Figure 1A), which corresponds to a proportion of 0.3–4.8% of archaeal 16S rRNA genes. Significantly higher ratios were found in the rhizosphere of the subsoil (*P* < 0.001), but no differences were observed between topsoil and subsoil within each soil compartment. The results did not change, when 16S rRNA gene abundance was corrected for the varying 16S rRNA gene copy numbers per genome (data not shown).

### Comparison of archaeal and bacterial richness and diversity in different soil compartments of top- and subsoil

The used barcoding approach resulted after de-multiplexing in 81388 (archaea) and 160768 (bacteria) flowgrams. After data trimming and de-noising, 80,151 and 158,567 high quality reads with an average length of 568 and 545 bp were obtained for archaea and bacteria, respectively. 8.1 and 1.3% of the reads were removed as chimeric sequences from the archaeal and bacterial dataset. One thousand and twenty-two sequences derived from chloroplasts in the bacterial dataset and were not included in downstream analysis. Also unknown sequences (21 archaeal and 3 bacterial reads) were not further processed. Sequences were analyzed on the level of 90 and 95% similarity and subsampled according to the minimum sample size in each dataset. Singletons were not excluded from the analysis, as they were not evenly distributed across the samples and variation between the six soil compartments exceeded the overall variation (Figure S2).

Richness of bacteria and archaea was estimated on a level of 90% similarity, where coverage was highest and expected effects of singletons derived from sequencing errors were lowest (Figure S3). Overall, bacterial richness and diversity was significantly higher compared to archaea. Interestingly, rarefaction curves showed significant higher richness in the topsoil for bacteria (*P* < 0.001), but a higher richness in the subsoil for archaea (*P* = 0.001). Nevertheless, Shannon diversity indices were for both, archaea and bacteria, higher in the topsoil (*P* = 0.026, *P* < 0.001; Figures 1B,C, Figure S4). In the subsoil only for bacteria differences between the soil compartments were found. In the drilosphere the highest diversity based on the Shannon index was observed (*P* = 0.009). Interestingly in this compartment the effect of soil depth for both archaea and bacteria was lowest.

### Soil depth and compartment-specific microbes as revealed by community composition

**Figure 2:**
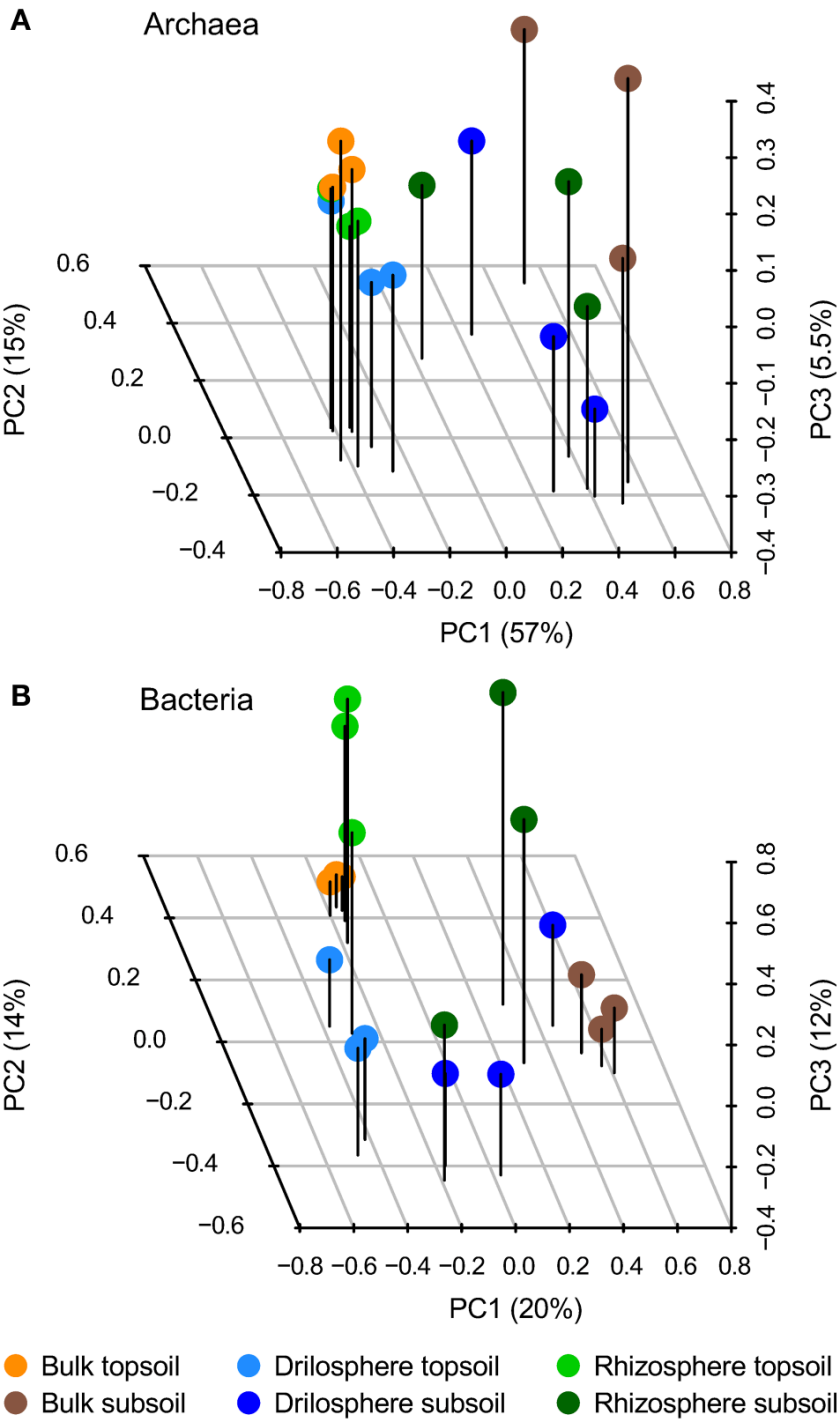
**Principal component analysis (PCA) of archaeal (A) and bacterial (B) OTUs based on 16S rRNA gene amplicons at 95% similarity level**. The first three components from the PCA of relative, Hellinger-transformed data are shown.

At 95% similarity level, clustering revealed 614 archaeal and 9425 bacterial OTUs. They were analyzed in the first step by PCA to investigate how the six compartments differ in their community compositions: the first three components are plotted in Figure 2. A clear separation between topsoil and subsoil could be detected for archaea only (PerMANOVA: *P* = 0.001), whereas the compartments bulk soil, drilosphere and rhizosphere showed no significant differences (*P* = 0.489). In contrast to these findings, a clear difference for compartments as well as for soil depth (*P* = 0.001, each) was found for bacteria. Variation between the replicates was lowest in bulk samples from topsoil for both, the bacterial and archaeal dataset.

**Figure 3:**
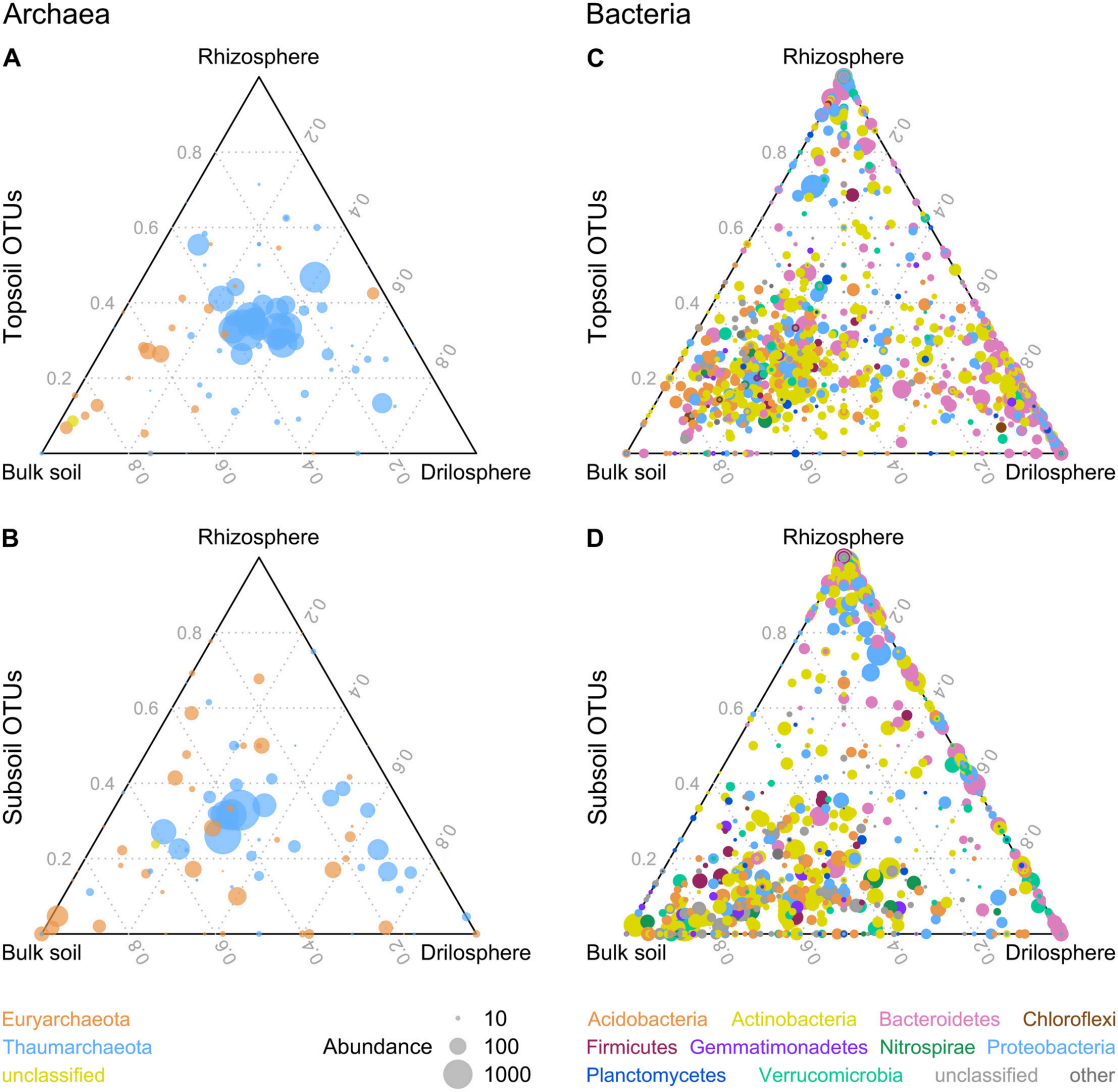
**Distribution of archaeal (A,B) and bacterial (C,D) OTUs between soil compartments in ternary plots**. OTUs with a higher abundance in topsoil or subsoil are displayed in **(A,C)** or **(B,D)**. Similarity level is 95% and only OTUs with a minimum absolute abundance of 5 are shown. The size of the dots represents the absolute abundance of one OTU.

Archaeal communities were dominated by the genus *Nitrosophaera* with a relative abundance of 90–99% for all six compartments (Figures S5, S6). This genus is known as an ammonium-oxidizing archaeon and the only abundant genus found in our dataset for the phylum Thaumarchaeota. Some OTUs classified as *Nitrososphaera* were significantly higher abundant in topsoil (9), whereas others dominate in subsoil (4). Euryarchaeota was the second phylum detected, being higher abundant in subsoil (*P* = 0.048). Most OTUs belonging to Euryarchaeota could not be classified further, except the methanogen *Methanosarcina* which was significantly higher abundant in the topsoil (*P* = 0.001) especially in the drilosphere and rhizosphere with >0.3% of all reads, whereas relative abundance in subsoil was lower (0.06%). Ternary plots for archaeal OTUs indicated for top- and subsoil (Figures 3A,B) that drilosphere and rhizosphere did not harbor “specialized” OTUs, which would be located at the respective tip area of the ternary plot. Only for bulk soil, specialized archaea were found, when top- and subsoil were compared, which were classified as Euryarchaeota. However, the majority of the archaeal OTUs was located at the middle of the ternary plot, harboring mainly Thaumarchaeota including 20 ubiquitous OTUs (all *Nitrososphaera*), that were present in all samples and contributed to 56% of the reads analyzed.

The bacterial community analyses revealed 21 bacterial phyla present, although only for 10 phyla relative abundance in all six investigated soil compartments was >0.5% (Figure S7): Actinobacteria (29–43%), Bacteroidetes (5–32%), Proteobacteria (10–24%), Acidobacteria (4–18%), Verrucomicrobia (3.4–5.6%), Planctomycetes (2–3.9%), Nitrospirae (0.3–3.5%), Firmicutes (1.5–2.8%), Gemmatimonadetes (0.4–2%), and Chloroflexi (0.2–0.9%). Acidobacteria, Gemmatimonadetes, and Planctomycetes were significantly higher abundant in bulk soil. In contrast to bulk soil, rhizosphere and drilosphere harbored a higher portion of Bacteriodetes. Proteobacteria in turn were typically found as major parts of the rhizosphere community. Besides the compartment type, also depth related differences were present on the phylum level. For topsoil only the low abundant phylum Chloroflexi was significantly increased, whereas in subsoil samples bacterial community harbored more Actinobacteria, Nitrospirae and Verrucomicrobia. For the latter phylum only for bulk soil and drilosphere significant differences were found. In addition, unclassified bacterial OTUs on phylum level (3–12%) were higher abundant in bulk samples from subsoil. Interestingly, Firmicutes did not show significant differences between the compartments. Data are summarized as ternary plots (Figures 3C,D).

**Figure 4:**
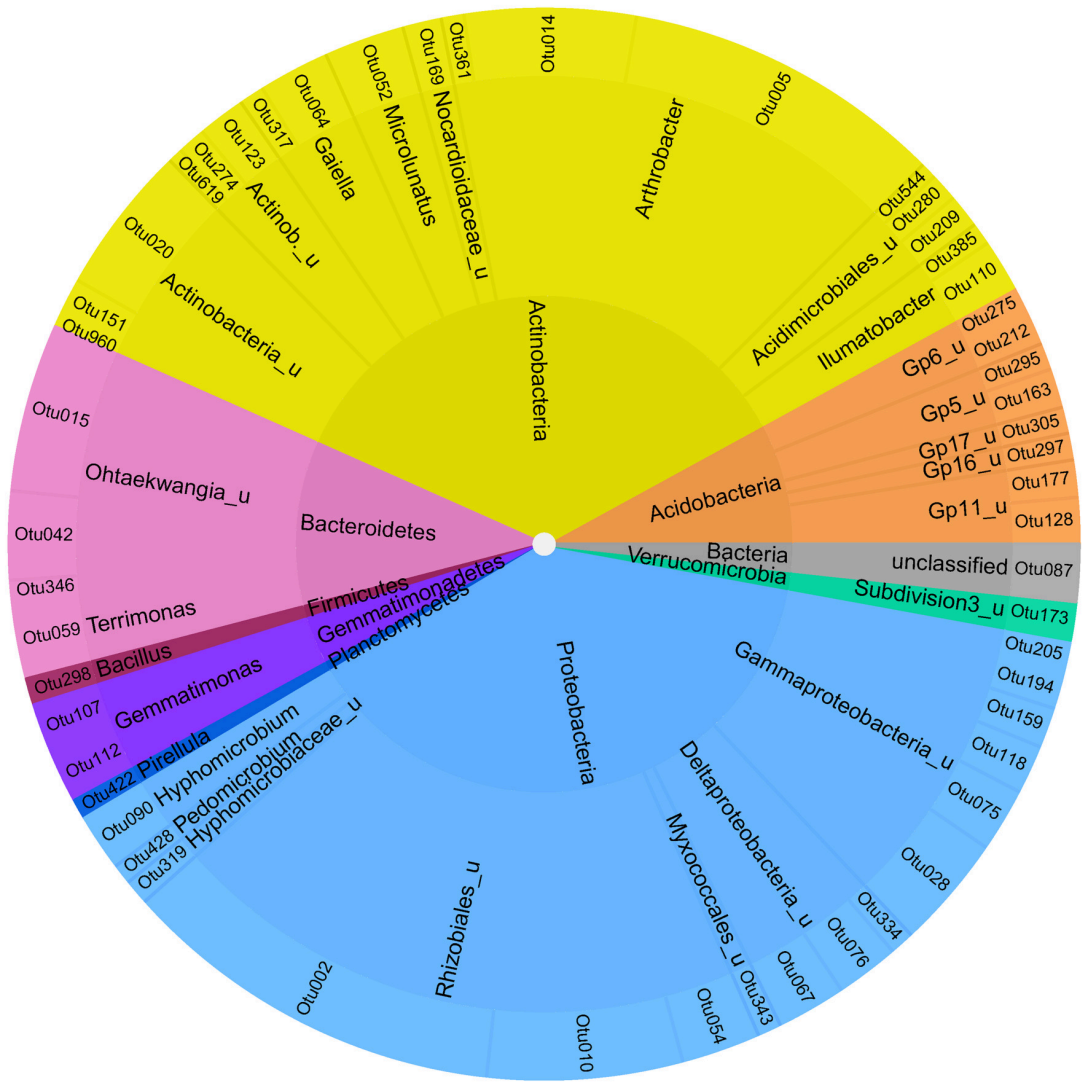
**Soil-intrinstic bacterial core microbiome**. Inner ring—phylum level; middle ring–next classifiable level—outer ring level of individual OTUs.

To identify a bacterial “intrinsic core microbiome” only OTUs at the level of 95% homology were selected, which were present in at least 2 of the 3 biological replicates for each of the six soil compartments and where the standard deviation did not exceed the mean value of the relative abundance to enable a low variation between the samples. This resulted in 52 both rare and abundant OTUs, that contributed in sum to 7.6% of the reads analyzed (Figures 4, S5). All abundant phyla were represented in the core microbiome with the majority of Actinobacteria and Proteobacteria accounting for 35 and 39% of the reads, respectively.

Besides the overall core microbiome, the core microbiomes were analyzed separately for topsoil and subsoil using the same criteria as described above. The bacterial topsoil core microbiome shared 4.3% of all OTUs between bulk soil, drilosphere and rhizosphere, which corresponds to 27% of the reads from the topsoil. In contrast, the bacterial subsoil core microbiome shared only 2% of OTUs, which accounted 16% of the reads. The same procedure for the archaeal dataset revealed an increased core microbiome as compared to bacteria both in topsoil and subsoil, but again, the subsoil archaeal core microbiome shared between bulk soil, drilosphere and rhizosphere (7% OTUs accounting for 69% of the reads) was smaller than in topsoil, where 11% of the OTUs were detected in all compartments, which represented 93% of the reads.

### Clusters of co-occurring bacterial OTUs

**Figure 5:**
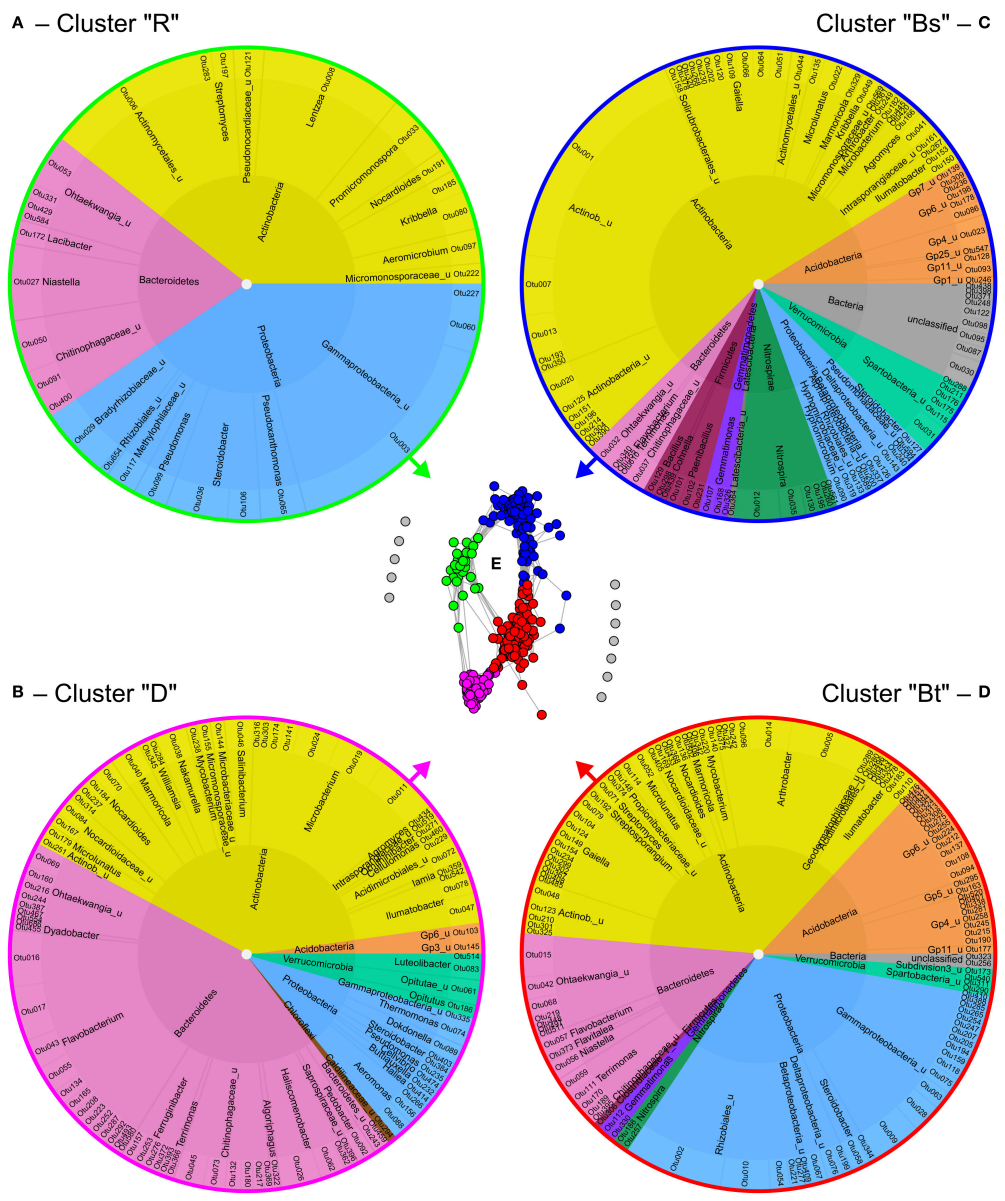
**Formation and composition of bacterial clusters of co-occurring OTUs (A–D) as revealed by network analysis (E)**. Each dot in the network represents one OTU at 95% similarity level and each connecting line a positive correlation with Spearman's rank correlation coefficient >0.6. For the gray colored OTUs in the network no positive correlations were found. Inner ring—phylum level; middle ring—genus or nearest classifiable level. R, rhizosphere; D, drilosphere; Bs, bulk subsoil; Bt, bulk topsoil.

Co-occurrence analysis of bacteria at 95% similarity level resulted in the identification of four clusters of co-occurring OTUs (Figure 5) that could be attributed to the different soil compartments and depths as revealed by clustering (Figure S8). In the dendrogram, replicates of bulk topsoil and bulk subsoil clustered closer together than samples obtained from rhizosphere and drilosphere, which emphasizes the high variability of those compartments. The clusters were further named “rhizosphere cluster R,” “drilosphere cluster D,” “bulk topsoil cluster Bt,” and “bulk subsoil cluster Bs” and reflect the significant differences found for the overall community composition between the six soil compartments (Figures 5A–D).

Remarkably, the clusters Bt and Bs shared 28 and 11 OTUs of the 52 OTUs of the core microbiome, respectively, but no OTUs were shared between the core microbiome and the hotspot clusters D and R. In the ternary plot, Bt and Bs clusters were located in the bulk soil-orientated middle area, whereas D and R clusters were more located at the tips of the triangle, where “specialized” OTUs were expected (Figure S9). Many phylogenetic lineages and genera were shared between the four clusters, although they were represented by different OTUs, especially *Nocardioidaceae* (Actinobacteria), *Ohtaekwangia* (Bacteroidetes), *Chitinophagaceae* (Bacteroidetes), and Gammaproteobacteria including *Steroidobacter*.

Bs clusters were characterized by the dominance of Actinobacteria (54%). Also low abundant phyla and unclassified bacteria were highly represented in Bs cluster. Acidobacteria were highly abundant in Bt and Bs clusters, and were represented by 4–6 classes. Interestingly, the four major lineages of Verrucomicrobia were restricted to one cluster each: Spartobacteria to Bs, Subdivision3 (Verrucomicrobiae) to Bt, and *Opitutus* (Opitutae) and *Luteliobacter* (Verrucomicrobiae) to D. Similar distribution pattern were observed for the phylum of Fermicutes: The genera *Bacillus, Cohnella*, and *Paenibacillus* were typical for the Bs cluster, whereas *Clostridiaceae* were part of the Bt cluster.

In contrast to the bulk soil clusters, D and R clusters harbored many specialized OTUs and lineages. Interestingly, drilosphere and rhizosphere shared more OTUs in subsoil (Figure 3D). The D cluster was dominated by Bacteroidetes with 43%. The R cluster was the smallest and harbored only Actinobacteria, Bacteroidetes and Proteobacteria. Especially the high proportion of Proteobacteria distinguished the R cluster from the others.

OTUs of a cluster that negatively correlated with most OTUs from other clusters, are listed in Table 1. In this respect the genera *Ilumatobacter, Gaiella, Marmoricola*, and *Steroidobacter* were of high interest. Each of these genera harbored different OTUs that are linked to different clusters and contributed strongly to the negative correlations between them. Acidobacterial OTUs distinguished the Bt and Bs clusters from each other as well as clusters D and R. *Flavobacterium*, again was a key genus in the D cluster that negatively correlated especially with OTUs from Bt and R clusters. *Aeromicrobium* accounted for most negative correlations of the R cluster with the Bt cluster.

**Table 1:** **Negative correlations between clusters of co-occurring OTUs**.

	**Cluster Bt**	**Cluster Bs**	**Cluster D**	**Cluster R**
Bt	-	Gp4_u Otu215; Gp6_u Otu394 *Marmoricola* Otu406 *Microlunatus* Otu114 Actinobacteria_u Otu048, Otu301 Ohtaekwangia_u Otu068 *Clostridiaceae*_1_u Otu306 *Steroidobacter* Otu344 Gammaproteobacteria_u Otu254	Gp6_u Otu275	*Ilumatobacter* Otu413 *Gaiella* Otu367
Bs	*Ilumatobacter* Otu267 *Agromyces* Otu041, Otu420 *Marmoricola* Otu329 *Gaiella* Otu066, Otu109 Bacteria_u Otu098, Otu248	-	Gp11_u Otu128 *Ilumatobacter* Otu153 *Gaiella* Otu120 Latescibacteria_u Otu364	Gp6_u Otu236 Actinobacteria_u Otu020 Bacteria_u Otu030, Otu371
D	*Microbacterium* Otu141 *Salinibacterium* Otu046 *Marmoricola* Otu040 *Nocardioidaceae*_u Otu167 *Flavobacterium* Otu017, Otu287, Otu580 *Aeromonas* Otu088 *Buttiauxella* Otu232 *Luteolibacter* Otu083, Otu514	*Ilumatobacter* Otu078 *Microbacteriaceae*_u Otu144 *Microlunatus* Otu179 *Ferruginibacter* Otu253 *Terrimonas* Otu366 *Chitinophagaceae*_u Otu073	-	*Flavobacterium* Otu016, Otu252, Otu493 *Ferruginibacter* Otu157, Otu253 Bacteroidetes_u Otu459
R	*Aeromicrobium* Otu097 *Streptomyces* Otu283	*Bradyrhizobiaceae*_u Otu029	*Steroidobacter* Otu106	-

*The most important OTUs of each cluster (row) responsible for minimal 20% of all negative correlations to another cluster (column) are listed. R, rhizosphere; D, drilosphere; Bs, bulk subsoil; Bt, bulk topsoil*.

## Discussion

### Variation within soil compartments on the plot scale

As shown in the PCA (Figure 2), the variation of both archaeal and bacterial communities was much lower in topsoil than in subsoil. The more homogeneous topsoil on the plot scale is a result of plowing and the high root density at the time point of sampling. Furthermore, overall the higher nutrient status in topsoil compared to subsoil might have induced lower gradients between the soil compartments. Thus, the variation between and within the soil compartments in subsoil were increased as a result of longer distances between hotspots and less disturbance from outside. These observations differ from non-managed ecosystems. For example, Eilers et al. (2012) showed a higher variation in topsoil compared to subsoils, when microbial communities of a forest soil where compared. Overall the drilosphere and rhizosphere communities in general shared more abundant OTUs in the subsoil as compared to the topsoil (Figure 3D). A possible explanation is that roots grow into earthworm burrows and *vice versa* earthworms invade biopores developed from decaying roots.

### Archaea—a small, but stable backbone of prokaryotic communities in the soil

Archaea were in all analyzed samples part of the soil prokaryotic community independent from spatial heterogeneity and depth. Their proportion in this study compared to bacteria is comparable to other studies where the microbiome of bulk soils has been analyzed (Bates et al., 2011; Pereira e Silva et al., 2012). Although their abundance based on 16S rRNA gene copies was below 5% in all samples, the highly abundant genus *Nitrososphaera* was a core genus and most likely strongly relevant for nitrification, as no OTUs indicative for ammonium oxidizing bacterial genera like *Nitrosomonas, Nitrispina*, or *Nitrosococcus* were identified. Especially in subsoil the dominance of only a few *Nitrososphaera* OTUs reflected the higher richness of archaeal communities as compared to topsoil. The common occurrence of *Nitrososphaera* in soils and their contribution to ammonium oxidation has been intensively investigated (Schauss et al., 2009; Tourna et al., 2011). A pronounced bias of the used archaeal 16S rRNA gene primers toward *Nitrososphaera* could be excluded, as the relation of the major archaeal taxa remained constant in a metagenome analysis after direct sequencing of the same soil samples (data not shown).

In the more oligotrophic environments of bulk soil, overall more archaeal 16S copies were detected and in particular unclassified OTUs from the Euryarchaeota increased. This points to an overall oligotrophic strategy of Euryarchaeota and is backed up by the higher archaeal richness which was observed in subsoil samples. Only the anaerobic methanogenic archaeon *Methanosarcina* was found in the copiotrophic environments of drilosphere and rhizosphere topsoil. As a residue of earthworm activity, the origin of this prokaryote might be the gut microbiome of invertebrates. However, also the assimilation of straw-derived carbon in the rhizosphere was shown for *Methanosarcina* (Shrestha et al., 2011) making it quite likely that microbes from the earthworm gut can survive in soil.

### The soil intrinsic core microbiome of bacteria

The definition of a core microbiome is still a challenging task (Shade and Handelsman, 2012). A “soil core microbiome” can be found by comparison of different soils, but this often neglects the spatial heterogeneity both on the horizontal and vertical axis and only gives information about the specific soil compartment investigated. Therefore, the attempt here was to identify an “intrinsic soil core microbiome” that can be interpreted as a backbone of a specific soil type, regardless of its depth and spatial heterogeneity. This study gives evidence that on phylum level the cluster Bt, mostly affiliated to the bulk topsoil, is indeed a good representation of the soil intrinsic core microbiome over horizontal and vertical gradients as it includes most phyla and groups found generally in soils (Stroobants et al., 2014).

Co-occurrence analysis revealed a well-defined microbial cluster in subsoil which clearly differs from the other clusters. The actinobacterial dominance in the Bs cluster suggests a high potential for secondary metabolism in subsoil that needs to be investigated further, as over 50% of the Actinobacteria could not be further classified. Their potential for plant growth promotion, mainly biocontrol of phytopathogens, (Haesler et al., 2014; Hamedi and Mohammadipanah, 2014) as well as for the degradation of recalcitrant carbon, which is typically found in deeper soil layers (Rumpel and Kögel-Knabner, 2011) might be immense. The abundance of Acidobacteria, which are reported as slow-growing microbes (Foesel et al., 2014), as well as members of Actinobacteria, Chloroflexi, and Gemmatimonadetes (Zhang et al., 2003; Davis et al., 2011), and endospore-forming Firmicutes in this cluster might explain the lower microbial activity in subsoil (Kramer et al., 2013; Stone et al., 2014; Uksa et al., 2015). The genus *Nitrospira* which was highly abundant in the Bs cluster is the possible complement to *Nitrososphaera* for the nitrification processes in this soil compartment, as no other known nitrite-oxidizing bacteria like *Nitrobacter, Nitrospina*, or *Nitrococcus* could be detected.

The co-occurring OTUs which were typical for drilosphere and rhizosphere indicated specialized microbial clusters with low overlaps to the bulk soil clusters. These OTUs could not been clustered according to soil depth like shown for the bulk soil (Figure 2), probably due to their vertical expansion in the biopores and nutrient input via earthworm cast and root exudates. The relatively high accessibility of nutrients therefore favors copiotrophic microbes and those interacting with earthworms and plants.

The high abundance of Bacteroidetes has been found in the driolosphere cluster, which can be explained nicely by the high abundance of this group of bacteria in the gut microbiome of invertebrates and earthworm cast (Furlong et al., 2002). Besides Bacteroidetes, also Proteobacteria are an essential part of the earthworm associated microbiome, like the genus*Aeromonas* which was specifically detected in earthworm cast (Kumari et al., 2012).

In the rhizosphere, a high interaction of Proteobacteria is well accepted (Berg and Smalla, 2009; Hartmann et al., 2009; Haichar et al., 2012; Lundberg et al., 2012). Those bacterial groups are more copiotrophic and able to grow fast on easy available nutrient (Fierer et al., 2007, 2012; Thomas et al., 2011).

Some antagonistic relationships which can be found in literature were confirmed in this study by negative correlations (Casida, 1983) and pointed out that not only nutrient availability but also the origin are relevant. Furthermore, OTUs from the same lineage or even the same genus (e.g., *Gaiella, Steroidobacter, Ilumatobacter, Ohtaekwangia*) are found to be negatively correlated and are therefore members to different clusters. These findings indicate antagonistic interaction or competition also on the species or ecotype/strain level and the presence of redundant phylogenetic lineages within differing soil compartments.

## Conclusion

In this study, pronounced differences in bacterial and archaeal community structure in relation to soil depth and hotspots have been described. We identified an intrinsic soil core microbiome, that shows high similarity to the bulk topsoil cluster, which is frequently analyzed in studies, where samples from different compartments are taken together or homogenized However, specific soil communities and phylogenetic lineages become visible at different depths or hotspots, when sampling was performed at smaller scales without mixing or homogenization of different compartments. These observed differences could berelated to the nutrient availability, nutrient quality (Fuka et al., 2008) and the presence of soil invertebrates or plants. However, this study is based on one time point during the vegetation only and one soil type. It must be clarified in future studies whether the observed response pattern is also valid in different soil profiles, e.g., sandy soils, and at other time points of plant growth, or at phases were plant residues in terms of litter or moisture regimes play a major role in soil carbon dynamics.

We could confirm that more putative copiotrophs are present in the hotspots like rhizosphere amd drilosphere as compared to bulk soil and that the proportion of putative oligotrophs increases mainly in bulk soil. Furthermore, the nutrient rich hotspots drilosphere and rhizosphere form distinct bacterial communities with many putative antagonistic interactions. As expected, the size of the archaeal core—microbiome shared between the soil different soil compartments is larger as compared to the bacterial core—microbiome, which indicates a lower specialization of archaea toward copiotrophic lifestyle. However, we could also show that in subsoil the shared microbiomes between bulk soil and the hotspots decreased.

Supported by enzyme studies (Uksa et al., 2015) and culture-based approaches (Maloney et al., 1997), oligotrophic organisms might be functional important for the turnover of recalcitrant material in the bulk subsoil, whereas in hotspots copiotrophic microbes contribute to the rapid decay of fresh organic matter. Therefore, the question arises, how oligotrophic and copiotrophic microbial patches in the subsoil interact with each other.

A general ecological classification of microbes based on the phylogeny however cannot be made, despite some bacterial phyla are mainly considered as oligotrophs or copiotrophs as they show correlation mainly with the carbon content (Fierer et al., 2007). In fact, the copy numbers of 16S rRNA genes per genome, which can vary highly within bacteria and archaea, is a better indication for the microbial lifestyle, as copiotrophic prokaryotes have the tendency to harbor more 16S rRNA gene copies compared to slow-growing organisms (Stoddard et al., 2014). Thus, the spatial distribution of microbes postulated to have an oligotrophic or copiotrophic lifestyle in this study must be confirmed on the basis of metagenome, metatranscriptome, and metabolome studies in the future.

### Conflict of interest statement

The authors declare that the research was conducted in the absence of any commercial or financial relationships that could be construed as a potential conflict of interest.
