# Supplementary figures and images for: Prokaryotes in Subsoil—Evidence for a Strong Spatial Separation of Different Phyla by Analysing Co-occurrence Networks
Source: Front Microbiol. 2015 Nov 18;6:1269. doi: 10.3389/fmicb.2015.01269 (PMC4649028; doi:10.3389/fmicb.2015.01269)

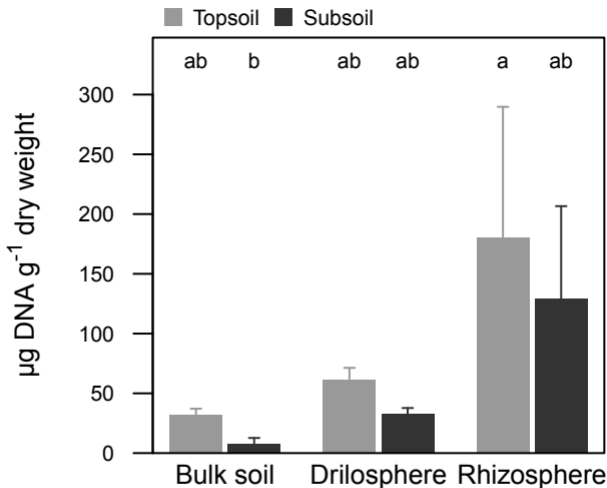

**Figure S1:** Biomass in different soil compartments and depth layers as estimated by DNA content.

Supplement: Supplementary file 1 [file Image1.PDF]

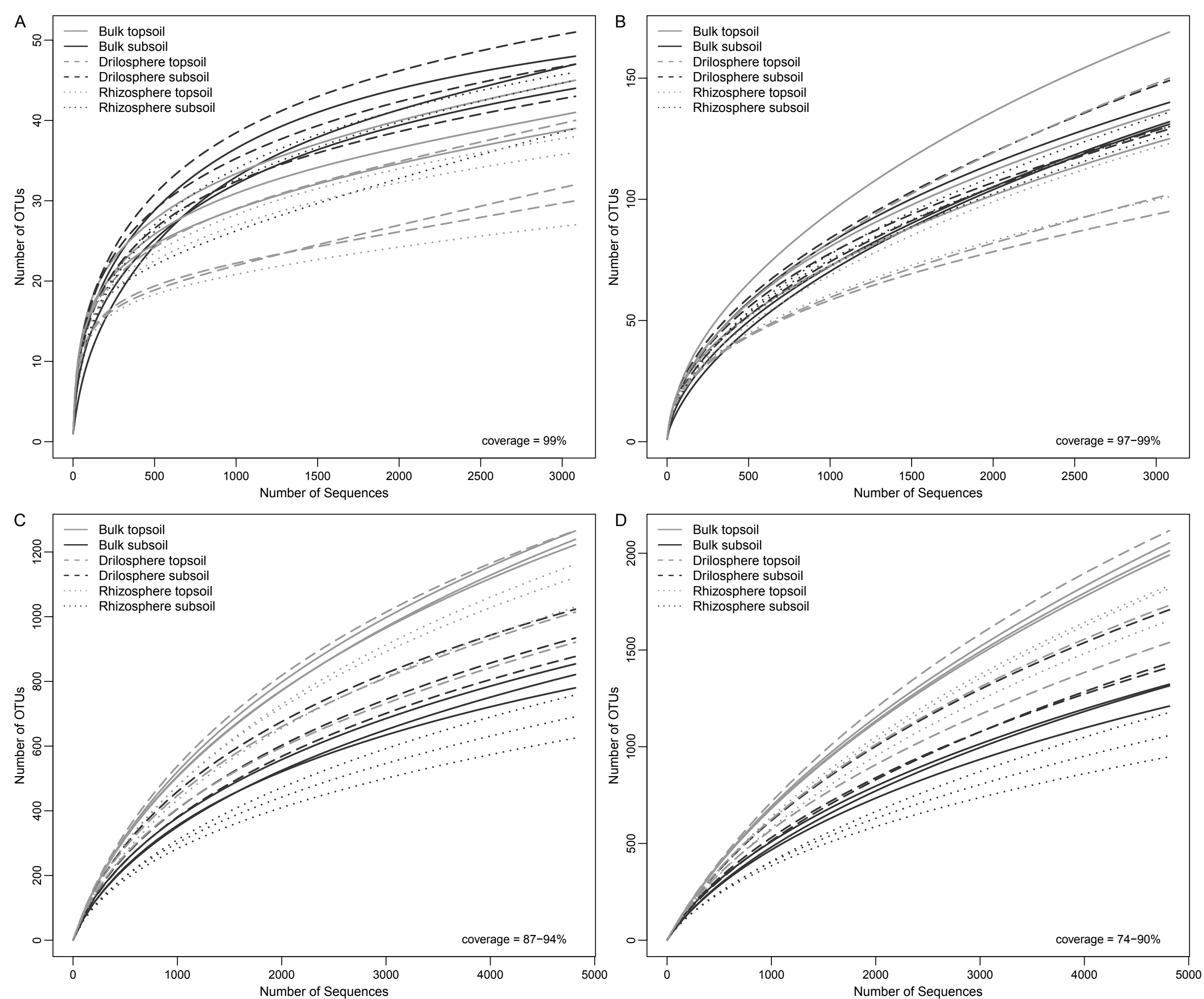

**Figure S3:** Rarefaction curves of archaeal (A, B) and bacterial (C, D) OTUs at 90% (A, C) and 95% (B, D) similarity level.

Supplement: Supplementary file 3 [file Image3.PDF]

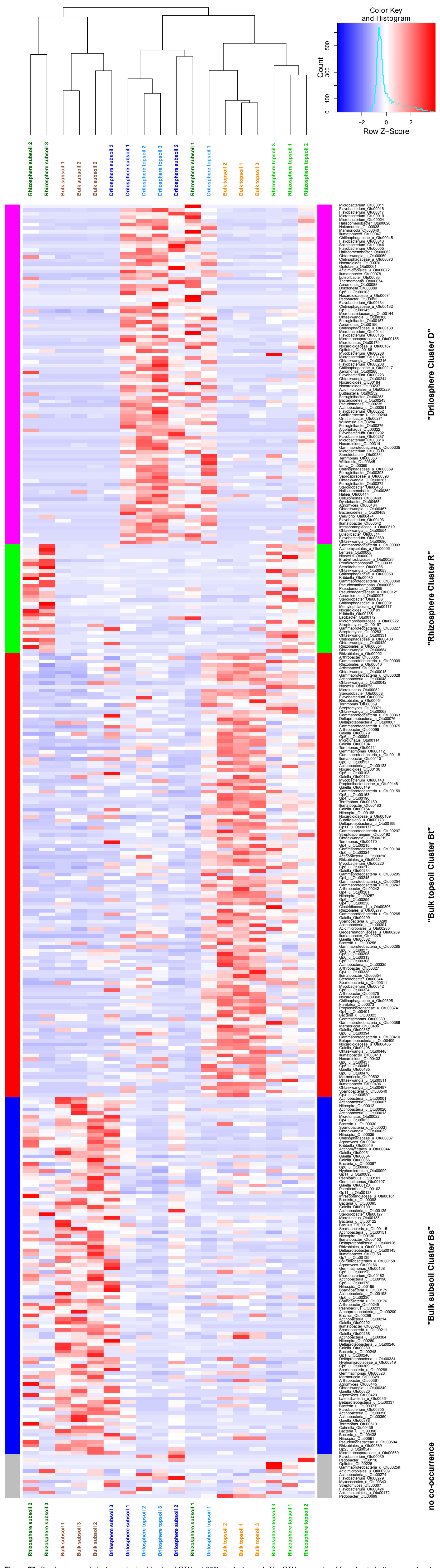

Supplement: Supplementary file 8 [file Image8.PDF]
