# Supplementary material for: Prokaryotes in Subsoil—Evidence for a Strong Spatial Separation of Different Phyla by Analysing Co-occurrence Networks
Source: Front Microbiol. 2015 Nov 18;6:1269. doi: 10.3389/fmicb.2015.01269 (PMC4649028; doi:10.3389/fmicb.2015.01269)
Supplement: Supplementary file 2 [file Image2.PDF]

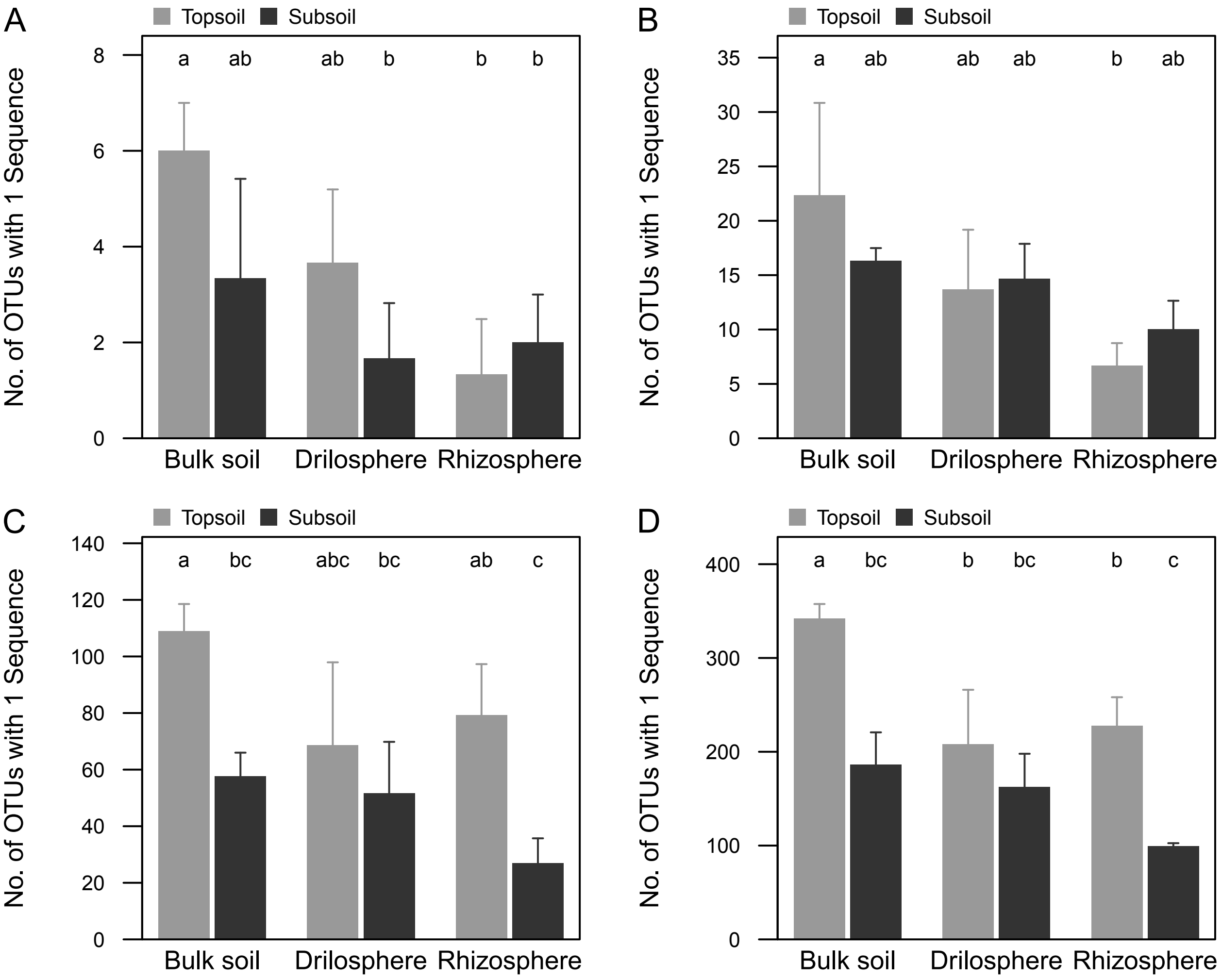

Figure S2: Count of singletons from archaeal (A, B) and bacterial (C, D) OTUs at 90% (A, C) and 95% (B, D) similarity level. Different letters indicate significant differences ($P \leq 0.05$).
