# Supplementary material for: Prokaryotes in Subsoil—Evidence for a Strong Spatial Separation of Different Phyla by Analysing Co-occurrence Networks
Source: Front Microbiol. 2015 Nov 18;6:1269. doi: 10.3389/fmicb.2015.01269 (PMC4649028; doi:10.3389/fmicb.2015.01269)
Supplement: Supplementary file 4 [file Image4.PDF]

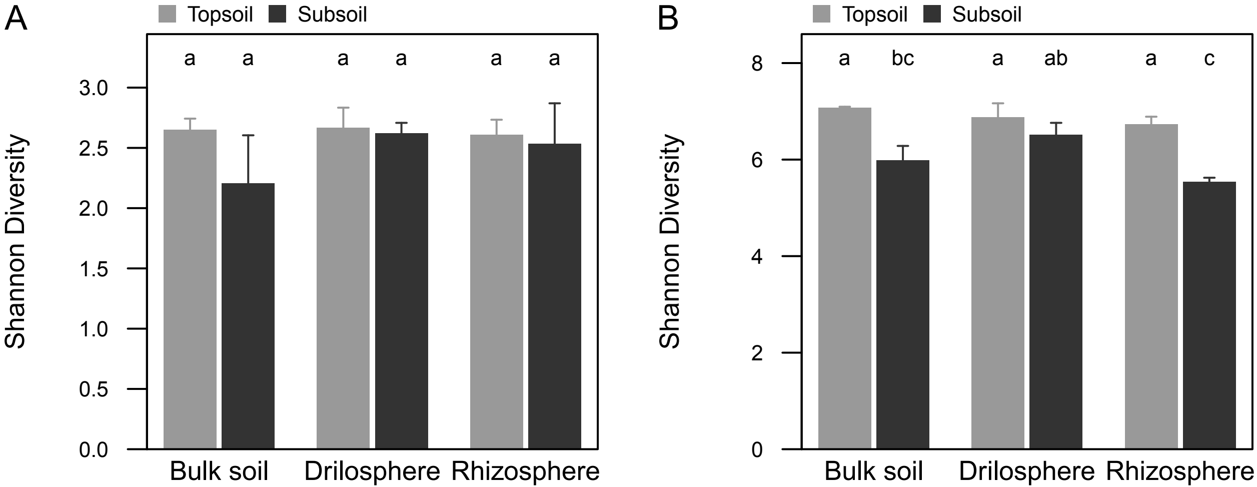

Figure S4: Shannon diversity index at 95% similarity level of archaea (A) and bacteria (B) in soil compartments of topsoil and subsoil. Different letters indicate significant differences ($P \leq 0.05$).
