# Supplementary material for: Prokaryotes in Subsoil—Evidence for a Strong Spatial Separation of Different Phyla by Analysing Co-occurrence Networks
Source: Front Microbiol. 2015 Nov 18;6:1269. doi: 10.3389/fmicb.2015.01269 (PMC4649028; doi:10.3389/fmicb.2015.01269)
Supplement: Supplementary file 5 [file Image5.PDF]

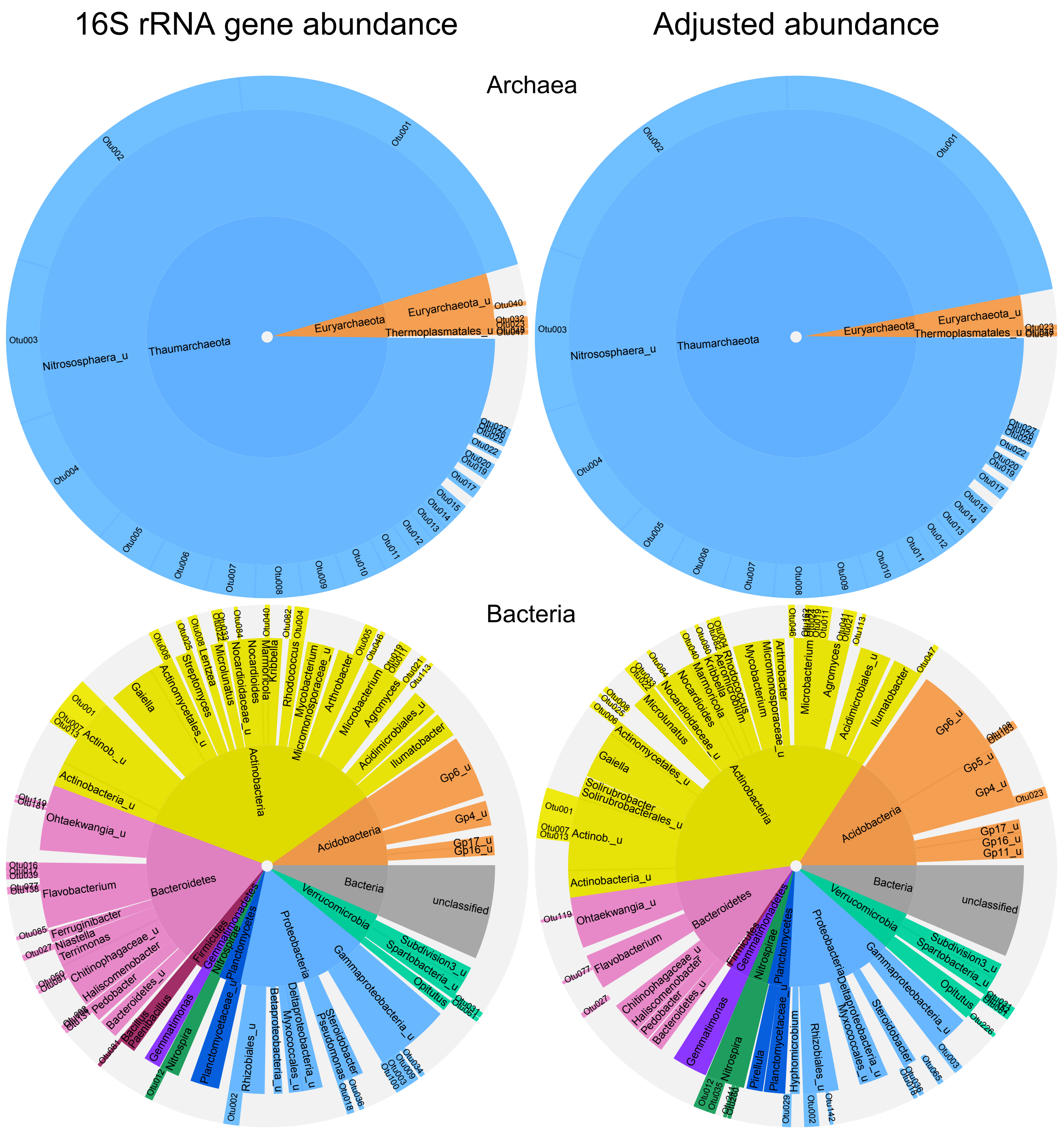

Figure S5: Estimation of the real abundance of archaea and bacteria from 16S rRNA gene copy numbers over all samples. Adjustment of the abundance was performed by information of varying 16S rRNA gene copy numbers per genome at *rrn*DB. OTUs were clustered at 95% similarity level and only OTUs with a relative abundance ≥ 1.5% in at least one of all samples are displayed. Inner ring - phylum level; middle ring - genus or nearest classifiable level.
