# Supplementary material for: Prokaryotes in Subsoil—Evidence for a Strong Spatial Separation of Different Phyla by Analysing Co-occurrence Networks
Source: Front Microbiol. 2015 Nov 18;6:1269. doi: 10.3389/fmicb.2015.01269 (PMC4649028; doi:10.3389/fmicb.2015.01269)
Supplement: Supplementary file 6 [file Image6.PDF]

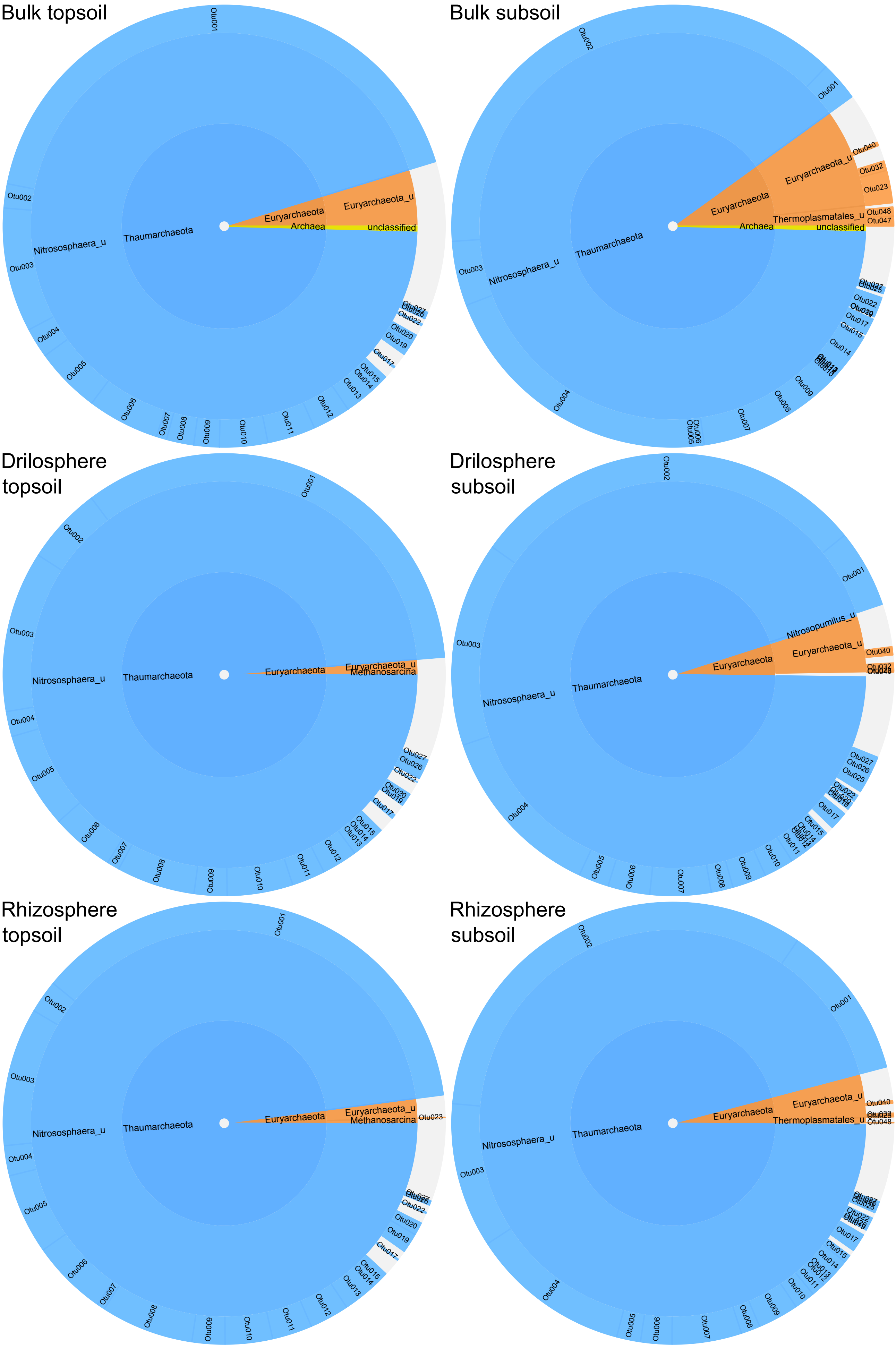

Figure S6: Archaeal community composition in different soil compartments of topsoil and subsoil. OTUs were clustered at 95% similarity level and only OTUs with a relative abundance ≥ 1.5% in at least one sample are displayed. Taxonomic groups below a relative abundance of 0.2% within each pie are not colored. Inner ring - phylum level; middle ring - genus or nearest classifiable level.
