# Supplementary material for: Prokaryotes in Subsoil—Evidence for a Strong Spatial Separation of Different Phyla by Analysing Co-occurrence Networks
Source: Front Microbiol. 2015 Nov 18;6:1269. doi: 10.3389/fmicb.2015.01269 (PMC4649028; doi:10.3389/fmicb.2015.01269)
Supplement: Supplementary file 9 [file Image9.PDF]

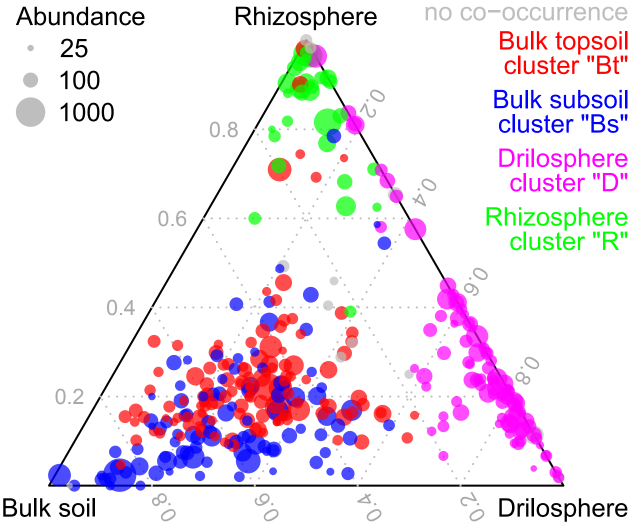

Figure S9: Distribution of bacterial OTUs between soil compartments and their affiliation to clusters of co-occurring OTUs according to cluster analysis at 95% similarity level.
